# Commentary: Clinical utilization of genomics data produced by the international *Pseudomonas aeruginosa* consortium

**DOI:** 10.3389/fmicb.2016.00770

**Published:** 2016-05-23

**Authors:** Erica L. Fonseca, Ana Carolina P. Vicente

**Affiliations:** Laboratório de Genética Molecular de Microrganismos, Instituto Oswaldo Cruz, FIOCRUZRio de Janeiro, Brasil

**Keywords:** SPM-1, metallo-beta-lactamase, integrative and conjugative elements (ICEs), *Pseudomonas aeruginosa* genomics, clone SP/ST277, byciclomycin resistance, BCR-1

The robust study on *Pseudomonas aeruginosa* genome evolution, epidemiology, antimicrobial resistance mechanisms, and pathogenesis performed by Freschi et al. (2015) will offer a huge contribution to clinicians to improve the management of infections caused by this organism. Moreover, it is a big step toward the application of such genomic data to understand cystic fibrosis infections and to establish a powerful surveillance system in the clinical routine. This study also presented the first snapshot of the *P. aeruginosa* resistome by performing searches on 389 *P. aeruginosa* genomes using the Resistance Gene Identifier tool from the Comprehensive Antibiotic Resistance Database (CARD) (McArthur et al., 2013). The resistome comprises all genes that are direct or indirectly involved with antimicrobial resistance emergence, which includes intrinsic and acquired resistance genes, distinct resistance mechanisms as well as new determinants not yet identified in the clinical environment (Wright, 2007). Freschi and Colleagues have applied an interesting strategy to recover the resistome information, however, we would like to highlight here that some methodological limitations can compromise this type of screening due to the resistome complexity. For example, they included in the resistome screening three *P. aeruginosa* genomes (9BR, 19BR, and 213BR; Boyle et al., 2012) that belong to the pandemic high-risk clone SP/ST277 (Fonseca et al., 2015) however, some relevant resistance genes that characterize this clone were not identified. The *bla*_SPM-1_ gene codes for a metallo-β-lactamase that confers resistance to all β-lactams antibiotics, including carbapenems, which is the first line antibiotic class in the treatment of infections caused by Gram-negative bacteria. This gene characterizes the *P. aeruginosa* clone SP/ST277, being recognized as a genetic marker of this lineage. Recently, it was demonstrated that the *bla*_SPM-1_ is carried by the ICE_*Tn*4371_6061, which also harbors the byciclomycin resistance *bcr-1* gene (Fonseca et al., 2015). ICEs (Integrative and Conjugative Elements) are genetic mobile platforms that can have a major impact in the spread and resistance emergence (Carraro and Burrus, 2014). However, neither *bla*_*SPM-1*_ nor *bcr-1* resistance genes were identified by Resistance Gene Identifier tool as part of the 19BR, 213BR, and 9BR resistome. Similarly, the IslandViewer, a specific tool to identify genomic islands, was not able to identify the ICE_*Tn*4371_6061 in none of these three genomes. In order to verify whether this finding resulted from a bias imposed by any parameters used by Freschi et al. (2015) to perform the analyzes or if it was a methodological limitation of the approach used, we applied the Resistance Gene Identifier tool on one genome of clone SP/ST277, extensively studied by our group (PS106), as well as on 9BR, 19BR, and 213BR. A previous BLASTn search using ICE_*Tn*4371_6061 as query revealed its presence in these genomes (Fonseca et al., 2015) however, they were not recognized in our analysis using Resistance Gene Identifier tool, showing some limitations of this approach in the resistome mining. Therefore, we provided here an evidence of an underestimated scenario of the antimicrobial resistance potential, which could also affect the efficient establishment of antimicrobial therapy strategies in the clinical routine. In conclusion, in spite of the valuable contribution of Freschi's study, we would like to stress here that, although the bioinformatics comprehend an important tool to study the overall biology of microorganisms and to assess specific genomic information, they all have limitations that are ameliorated when distinct *in silico* approaches are performed.

## Author contributions

All authors listed, have made substantial, direct and intellectual contribution to the work, and approved it for publication.

### Conflict of interest statement

The authors declare that the research was conducted in the absence of any commercial or financial relationships that could be construed as a potential conflict of interest.
